# Constructing social identity through multiple “us and them”: a grounded theory study of how contextual factors are manifested in the lives of residents of a vulnerable district in Brazil

**DOI:** 10.1186/s12939-020-01196-2

**Published:** 2020-06-05

**Authors:** Natalia Vincens, Martin Stafström, Efigênia Ferreira, Maria Emmelin

**Affiliations:** 1 Division of Social Medicine and Global Health, Department of Clinical Sciences in Malmö, Lund University, Malmö, Sweden; 2 Occupational and Environmental Health, School of Public Health and Community Medicine, Institute of Medicine, University of Gothenburg, Gothenburg, Sweden; 3 CAPES Foundation, Ministry of Education of Brazil, Brasilia, DF Brazil; 4 Department of Social and Preventive Dentistry, Federal University of Minas Gerais, Belo Horizonte, Brazil

**Keywords:** Inequity, Social determinants of health, Social identity, Grounded theory

## Abstract

The association between contextual factors and health inequalities is well documented, also in Brazil. However, questions about how contextual factors actually affect health and well-being persist. The aim of this qualitative study was to explore how contextual factors—i.e., social stratification and neighborhood opportunity structures—are manifested in the lives of the residents of a vulnerable district in Brazil. We used a Constructivist Grounded Theory approach based on 12 in-depth interviews. The core category constructing social identity through multiple “us and them” is supported by eight main categories that characterize different pairs of “us and them”, based on internal and external aspects of the social processes involved. Our findings strengthen and support the links between contextual factors and health inequalities, highlighting the relevance of downward social comparison, territorial segregation and stigmatization and erosion of social capital in the construction of social identities and the manifestation of social hierarchies and neighborhood structures in the Brazilian context. Ultimately, these create shame and stress but also pride and empowerment, which are recognized determinants of health inequities.

## Background

Following the renewed interest in social determinants of health in the past decades, contextual factors have become increasingly relevant to the health equity debate [1]. The social determinants of health, comprising these contextual factors, have been defined by the World Health Organization as “the conditions in which people are born, grow, live, work and age” [2]. These conditions are shaped by the distribution of power and resources, generating health inequities—i.e., the unfair and avoidable disparities in health between socioeconomic groups. Ample evidence supports the association between various contextual factors and health inequities in different settings. However, questions about how these factors actually affect people’s health and well-being persist.

Brazil is one of the most unequal countries in the world and despite social improvements in the past 30 years, social and health inequalities remain [3, 4]. In the twentieth century, Brazil underwent rapid industrialisation while also facing political instability, with military takeovers and authoritarian regimes, intertwined with short interludes of democratic rule. Brazil has experienced its longest period of democracy (approximately 30 years) after it was reestablished in the mid-1980s. The country has three levels of autonomous government—federal government, 26 states and a federal district, and 5563 municipalities—structured with an independent judiciary, an executive branch led by the president, and a bicameral legislature. The Unified Health System (SUS) was created in 1988 based on the principles of universality, integrality, equity, and social participation. After initial and some still persistent challenges (e.g, insufficient funding) substantial decentralization was introduced and social participation in policymaking and accountability increased [5]. With SUS, healthcare utilization increased, and unmet healthcare needs lessened [6, 7]. Alongside this new health system, poverty declined, and overall living conditions improved. These improvements have been attributed to several social policies, with special attention to the program Bolsa Familia, the largest conditional cash transfer program in the world [8, 9]. Still, there are large disparities within the country in infrastructure and availability of public services—including healthcare access and utilization—between individuals, and across neighborhoods, states and regions. Following these disparities, health inequalities with regard to various health outcomes including access to healthcare are markedly evident [3, 5, 10, 11]. Furthermore, with the election of far-right president Jair Bolsonaro in 2018, a democratic crisis is unraveling [12], jeopardizing even more health and health equity in the country [13].

In this study we focus on two contextual factors: (i) social stratification and (ii) neighborhood opportunity structure [14]. These are relevant social determinants of health, in Brazil as elsewhere [15–19]. Social stratification, according to Bourdieu, is based on different capital—economic, cultural and social—that interact to place individuals in the social space. Within that social space social classes are thus distinguished and defined in relation to others, based on the shared circumstances of a given position and the distance between positions. Physical and social aspects of the neighborhood have been associated with health inequality [20–23]. These aspects constitute the local “opportunity structure”, i.e., “socially constructed and socially patterned features of the physical and social environment which may promote or damage health either directly or indirectly through the possibilities they provide for people to live healthy lives” [14].

The links from these contextual factors to health inequalities have been previously conceptualized based on the relative deprivation theory, for instance [17, 24–27]. In this theory, the relative differences in the socioeconomic position affects health. The emphasis is not on the absolute effect of resources but what individuals can achieve with their resources in comparison to others in society [26]. Further developments from relative deprivation to health have been articulated through (i) social comparison [24, 28, 29], (ii) social capital and (iii) territorial segregation/stigmatization, among other mechanisms [30]. These have been suggested to ultimately generate social stress, which would in turn be detrimental to health [24].

Social comparison theory, initially proposed by Festinger [31] and further developed by others [32, 33], has been used to understand the mechanisms from social inequalities to health and well-being [34]. Social comparison theory has for instance been used to discuss the relevance of the direction of the comparison—either upward or downward in the social hierarchy—the choice of the reference group and the repercussions of social comparison for the individual’s social identification, health and well-being [35–37]. Empirical evidence supports the association between relative deprivation and poor self-rated health based on the social comparison argument [38].

Social capital is generated in trustful and reciprocal social relations that result in varied social resources—e.g., social support and sense of belonging at individual-level and better government performance or social inclusion at collective-level [39, 40]. At individual-level social capital has been shown to function as a buffer in the association between social inequality and health [41]. At collective level (the social cohesion approach), evidence suggests an association between income distribution and social capital, supporting that egalitarian societies are more socially cohesive, with a positive social environment [42–44]. The erosion of social capital following larger social inequalities has been related to social stress, exclusion, isolation and hostility and poor health outcomes [26].

Studies on the effects of territorial segregation and stigmatization on health adds another relative dimension to the discussion about the effects of neighborhood deprivation on health [45–50]. These studies have highlighted the relevance of neighborhoods beyond the physical and social conditions *within* a geographical area, bringing the notion that neighborhoods, like individuals are positioned and further differentiated in the social space in relation to other areas. Wacquant, Slater [51] have, based on Bourdieu and Goffman, articulated the relevance of neighborhoods as symbolic spaces for the social and health inequalities discussion, as segregated neighborhoods both represent and reinforce inequalities, through territorial marginalization but mostly stigmatization.

In a previous study, investigating the association between the target contextual factors and health in Brazil, we demonstrated a synergism between income distribution and neighborhood basic infrastructure on self-rated health in Brazil [23]. Neighborhood basic infrastructure—i.e., access to water, sewage, electricity and garbage collection—amplified the effects of state-level income distribution on self-rated health. These findings highlighted the relevance of basic infrastructure in the association between income inequality and health and the interplay between social determinants of health at different levels. In the current study, we wanted to further understand the mechanisms and processes that could explain the observed synergism and the general mechanisms linking contextual factors and health. The aim was to qualitatively explore how social stratification and neighborhood opportunity structures are manifested in the lives of the residents of a vulnerable district in Brazil. The relative deprivation theory and the concepts of social comparison, social capital and territorial segregation/stigmatization were used as sensitizing concepts “suggesting directions along which to look without steering what to see” [52, 53].

## Methods

### Study design

We used a qualitative study design, following Charmaz’ Grounded Theory (GT) approach [53], aiming at developing a theory/model about the processes involved in how the targeted contextual factors are manifested in the lives of residents of a vulnerable urban setting in Brazil. We believe Charmaz approach to be appropriate because of its focus on the interpretative understanding of experiences, giving voice to the participants themselves. Individual in-depth interviews [54] were chosen to capture the participants’ narratives about what it means to live in a vulnerable area. Individual interviews were regarded most suitable to preserve the privacy and safety needed for participants to be able to talk openly about sensitive issues such as crime and drug dealings common to the study setting.

### Study setting

We performed the study in the municipality of Belo Horizonte, the state capital of Minas Gerais, in wealthy Southeast Brazil. Belo Horizonte has an estimated population of 2.513.451 inhabitants in 2016 (6th larger city in Brazil). It was built in 1890s as one of the first planned modern cities in the country, surrounded by and nowadays curbed in the mountains. Already in 1940s population growth disrupted the initial urban settlement. The ongoing growth and increasing urbanization process are seen as both a cause and a consequence of social inequalities in the city. In fact, Belo Horizonte is one of the most unequal cities in Latin America after São Paulo, Rio de Janeiro and Bogotá (ONU-HABITAT, 2012; UNDP 2013).

Belo Horizonte has 216 Especial Zones of Social Interest (ZEIS in Portuguese), comprising *vilas*, *favelas* and *public housing estates.* These *ZEIS* are characterized by having a low socioeconomic population and great need of urbanization projects. The terms vila and favela are used almost interchangeably in Belo Horizonte referring to the informal and irregular land settlements. In Belo Horizonte, these ZEIS correspond to 16,2% of the city population in only 5% of the city area, revealing the high population density that also characterizes these special zones. We purposively targeted one ZEIS within one district in Belo Horizonte to theoretically represent a vulnerable settlement common in urban Brazil, yet special for having been completely urbanized. Historically, this was an informal settlement (favela/vila) formed in this district around 1960, coexisting with the rest of the district ever since.

**Fig. 1:**
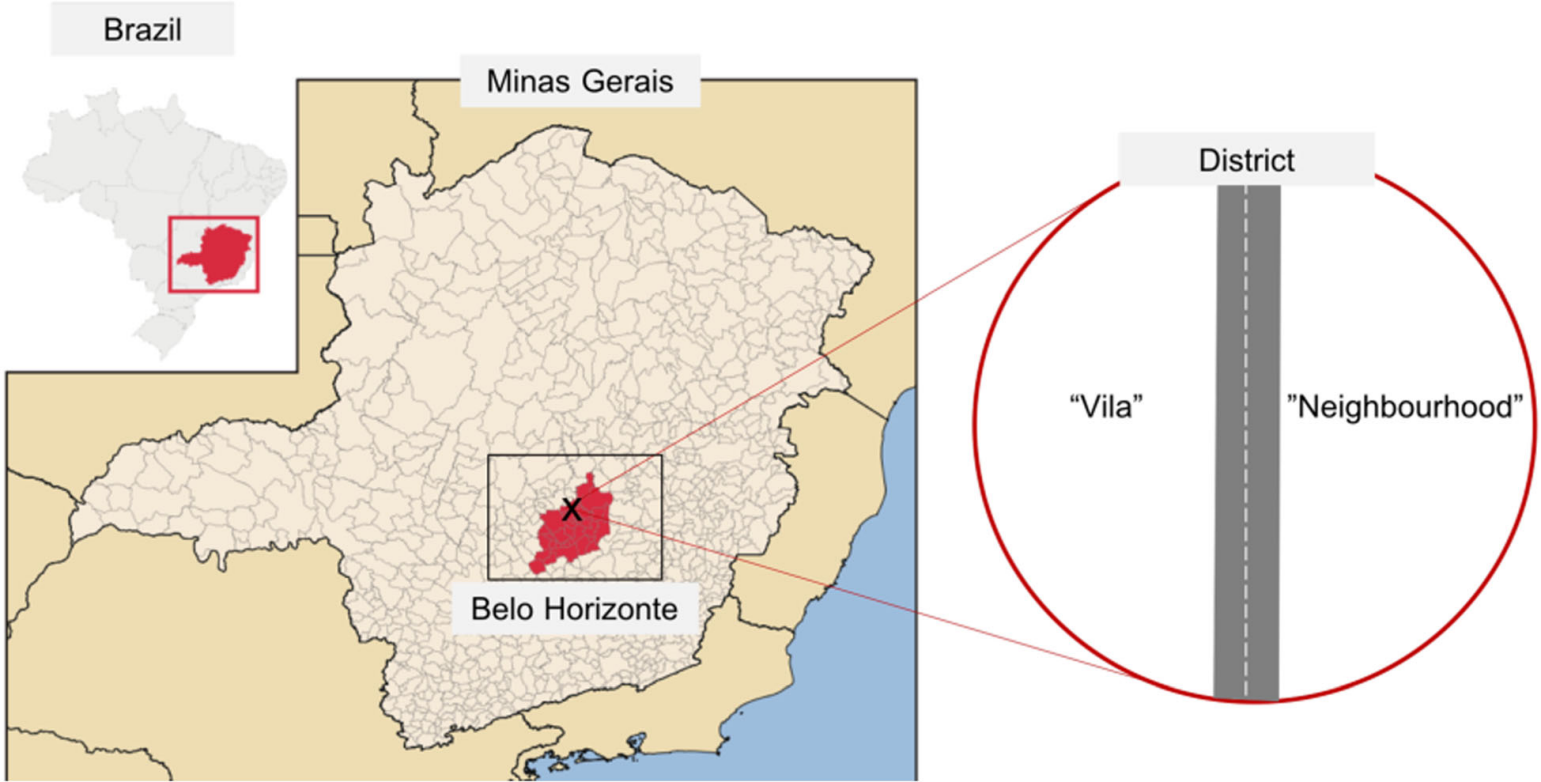
Map locating the study setting: a district in Belo Horizonte, Minas Gerais in southeastern Brazil. The district has “vila” and “neighborhood” sides. Map retrieved and adapted with permission from https://commons.wikimedia.org/wiki/File:MinasGerais_MesoMicroMunicip.svg. Copyright 2008 by Raphael Lorenzeto de Abreu

Since the year 2000 these special zones, including the one chosen for this study, have been targeted by projects aiming at the urbanization of vilas and favelas, focusing on better housing and overall basic infrastructure. Before the urbanization, vilas and favelas often suffered from floods, landslides and other consequences of the poor infrastructure. In these zones crime and violence are common and related to drug trafficking and frequent encounters with the police [55, 56]. For the studied ZEIS we will refer to the recently urbanized settlement as “vila” and the rest of the district as “neighborhood”, following the terms used by the local population (Fig. 1).

### Sampling of participants

We initially aimed to include residents from the vila because of their social vulnerability and high probability of poor health outcomes, considering their low socioeconomic positions and the physical and social characteristics of the area. Participants from the vila were recruited purposively to find a variation in experiences regarding social stratification and neighborhood structures, thus including men and women, in different age groups and educational backgrounds. Recruitment was done in collaboration with a community health worker placed at a local school. The community health worker facilitated the contact of the research team with the community members, introducing the researchers to the community. The participants were approached through their connection with the school—i.e., parents or other family members, students from the Adult Education Program, and school staff who lived in the district. The community health worker or the first author (NV) then contacted the participants, in person or via phone. Some people declined the invitation presumably because of fear of retaliation from drug dealers. Field visits and particularly memoing connected to the first couple of interviews indicated the relevance of living in a divided district and the emergence of a tentative category—the construction of us and them—in the manifestation of social hierarchies and neighborhood structures on the lives of those living in the entire district. This led us to broaden our sampling, following the theoretical sampling rationale, and thus intentionally add participants from the neighborhood side. This change in the sampling strategy led us to further develop the category and uncover the concept of “multiple” us and them. The content of the thematic interview guide and the process of sorting and integrating the analysis also changed to cater for new tentative ideas supporting the “multiple” us and them. Unfortunately, resource constraints (possibility to stay longer in the field) did not allow us to further develop the “multiple” us and them and pursue interviews with other groups such as the extreme poor/criminals in the vila or others’ outside the district. The sampling proceeded as described above and resulted in 12 in-depth interviews being performed. Instead of claiming saturation, following Charmaz recommendations [44], we strived to reach *theoretical sufficiency,* also by going back to the data, recoding and re-discussing emergent concepts as well as comparing the relationships within and between categories. In that way, we claim that these 12 in-depth interviews in connection to the field visits and memoing provided data with enough range, depth and nuance to support the development of the proposed model [57].

### Data collection

Data collection was preceded by field visits to the district with informal conversations with community members on both vila and neighborhood sides. Unfortunately, there was no safe opportunity to visit the inside parts of the vila. The actual interviews were conducted by the first author (NV), who is Brazilian, between June and July 2017. Interviews took place at the school facilities, which was considered a safe zone for both participants and researchers. Before the interviews, participants were informed about the objectives of the study. All participants accepted the invitation to come to the school and verbally agreed to be interviewed. The informed consent was signed later in connection with the interviews. For the interview we had prepared a thematic interview guide that was tested in a pilot interview, which resulted in the inclusion of seven broad themes, covering participants experiences and reflections on: district characteristics, physical environment, daily life, social relations, health and well-being and hopes for the future. The interview guide was flexible and the interviewer used open-ended questions and probing to capture participants’ experiences. The interviews took between 35 min and 1 and a half hours and were audio-recorded. The interviews were conducted in Portuguese, native language of the first author (NV) and all the participants.

### Analysis

**Fig. 2:**
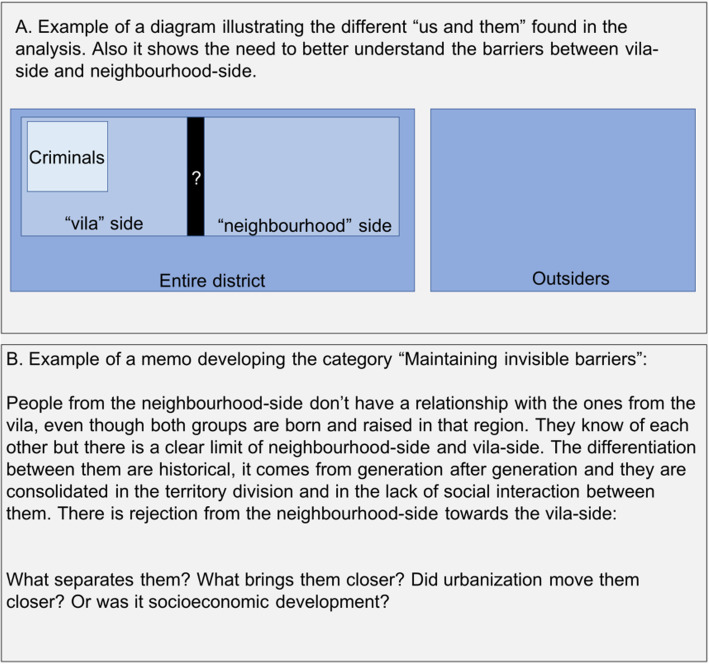
Examples of a diagram and a memo used in the analysis

The first author (NV) conducted the analytical process that started already during data collection, with memos and peer debriefing within the research group (ME and EF). First memos were written in connection with field visits and interviews. After all data were collected interviews were transcribed verbatim (in Portuguese). Coding, diagrams and memos were made mostly in English to allow participation of all research members in the analytical process. The first author (NV) was responsible for coding the text following Charmaz’s Constructivist Grounded Theory [53] with the stages of initial, focused and theoretical coding. Constant comparison with oscillation between data and categories characterized the analysis process and memos and diagrams assisted sorting, integrating and describing of the developed categories. The analysis resulted in the construction of a core category, eight main categories with supporting subcategories that further characterized the variation in the social processes in focus. Figure 2 provides an example of a diagram and a memo used in the development of the theoretical model of different “us and them” uncovered in the analysis and the initial elaboration about the emerging category “maintaining invisible barriers”.

### Ethical considerations

The study was approved by the Ethical Committee for Research, UFMG, Brazil (COEP-UFMG, license no. CAAE: 67810017.0.0000.5149–26/06/2017) and informed consent were obtained from all participants. NV informed participants about confidentiality, data security and, about their rights to withdraw from the study at any time. As a Brazilian, being from the city, the first author was aware of the social cues and rules of conduct regarding security, crime and violence. The school setting and support from the school staff provided a safe zone for the interviews which were held in private rooms within the school setting. To further preserve the security of participants we have omitted the name of the district and detailed information about the participants. Considering the sensitivity of the issues discussed and that criminal activities were disclosed during the interviews a senior researcher in the collaborating Brazilian university was prepared to assist participants with information about available support and reporting possibilities. For perpetrators of crime we considered: (i) the risks of breaking confidentially and loosing relevant data were greater than the risks of not reporting common and small criminal activities and (ii) the societal benefits of understanding the social phenomena in focus were greater than the benefits of reporting criminal activities, except in serious pending cases—although there were none—which we would have a legal obligation to report to the police.

## Results

Of our participants, six lived in the vila, four in the neighborhood, one has lived in both sides and one has lived in the vila but had recently moved out to a different district. The sample included five men and seven women and their age ranged from 22 to 62 years. Educational levels varied, with four participants having finished high school, two having completed primary school, four having incomplete primary school and two being illiterate.

### Constructing social identity through multiple us and them

**Fig. 3:**
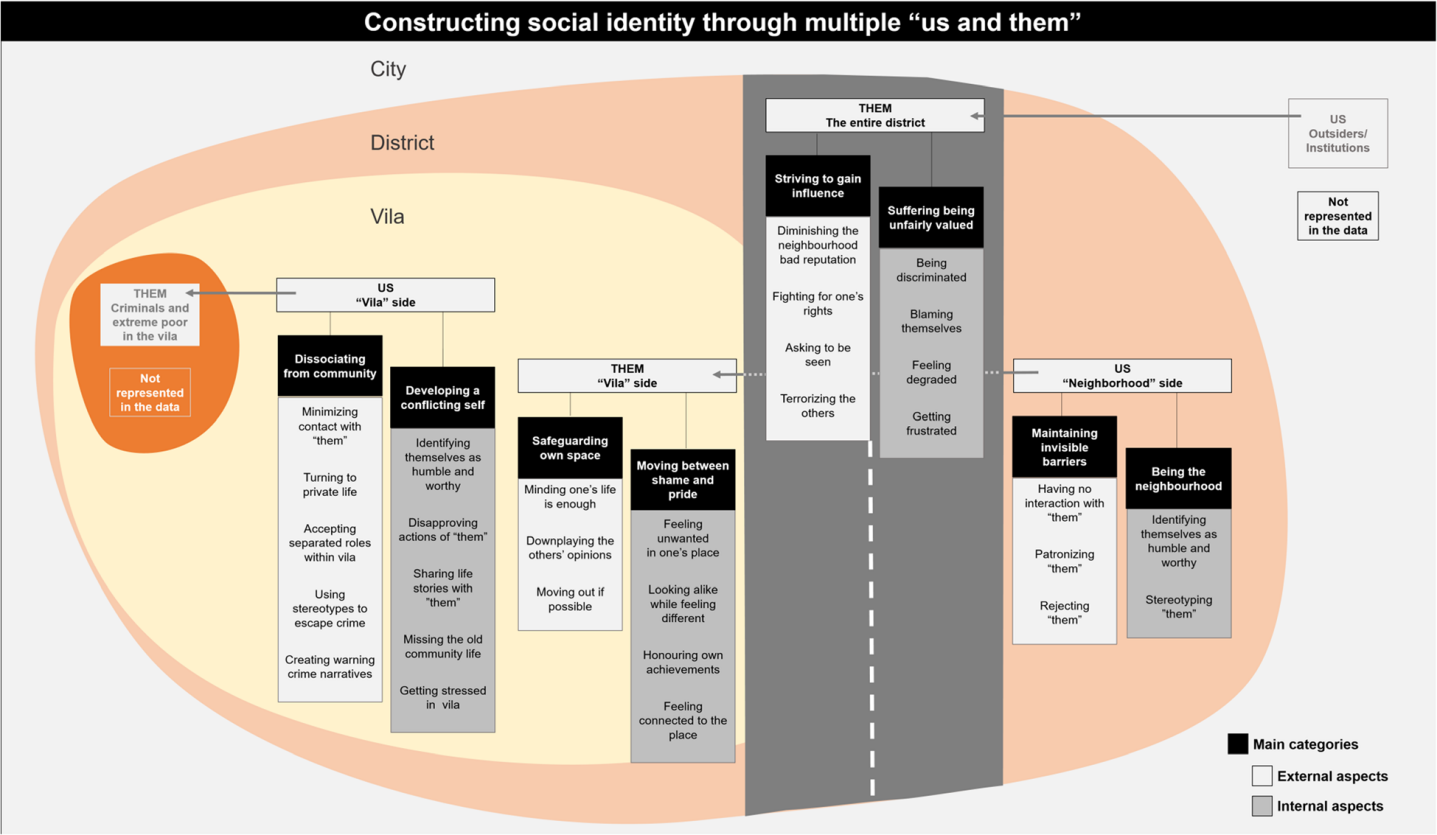
Theoretical model of internal and external aspects of the social identification process among residents of a vulnerable district in urban Brazil

We developed one core category “Constructing social identity through multiple us and them”, which describes how people in a vulnerable district socially situate themselves based on reference groups, social comparison and social position indicators, with varying influence on recognized determinants of health such as social stress and empowerment. Figure 3 gives an overview of the developed theoretical model. The eight main categories (white text with black background) illustrate external and internal aspects of the social processes related to the construction of social identity through each pair of “us and them” uncovered in the analysis. The subcategories (black text with lighter background) provide further characterization of the social processes indicating various strategies used in the construction of these social identities.

Residents of this vulnerable district position themselves socially based on within group similarities and between groups differences. In our model, we argue that these processes of social classification of both within and between groups social identities are marked by the presence of groups within groups. For the construction of our theoretical model, we used the direction of the most prominent othering process—which we have realized follows the power direction in the social spectrum—to label the “us” and the “them” perspectives in each pair of us and them: (i) within the vila residents assume the “us” perspective regarding the criminals and the extreme poor (them, not represented in the data); (ii) within the entire district residents from the neighborhood-side were labeled “us” and residents from the vila-side “them” and; (iii) between the entire district and the city, yet another us and them pair refers to the outsiders and the institutions (“us”, not represented in the data) and residents from the entire district (“them”), including now vila and neighborhood sides. Importantly, participants assume different perspectives in constructing social identity in relation to the different pairs of us and them. Participants from the neighborhood-side for example assume the “us” perspective in the interaction between vila and neighborhood while they become “them” together with residents from the vila in the interaction between outsiders/institutions and the entire district.

In the more detailed presentation of the results below we depart from the model and in the text our developed categories are given in bold with their supporting sub-categories and selected quotes from the interviews.

### A. "Us" perspectives

#### External aspects of the construction of social identity

Vila and neighborhood residents use different strategies to “separate” themselves from others in lower socioeconomic positions. While vila residents **dissociate themselves from community life**, neighborhood residents **maintain invisible barriers **between themselves and the ones across the road (the vila). Within the vila there was a history of greater proximity: vila residents, criminals and the extreme poor shared life-stories, living spaces and used to have regular encounters within the common areas of the vila. To create this group-divide within the vila, vila participants minimize contact with the others and turn to their private lives. Social interactions and support are increasingly based on family and extended family, who often live in the same residential area. The occupation of the common areas—that were previously shared by all groups in the old favela environment—are now marked by private use in the residential buildings, for instance as private parking spaces.

Especially in relation to criminals, participants from the vila are accepting separated roles, viewing criminals as having different social identities and practices. Still, in order to co-exist in the same space, they recognize the need and paybacks of accepting their separated roles within the vila. For instance, there is a sense of respect and reciprocity in the relationship between them: “*[The criminals] respect us. I mean, they respect to get respected”* (P9). The vila participants also mention a sense of security when in the vila, as criminals would not hurt and possibly even protect them: “*you can come and go. You won’t see robbery here. You won’t see no one confronting you”* (P10). However, respecting the criminals is not regarded easy since the vila participants felt forced to keep quite regarding criminal activities, fearful of retaliation: “*So, we try to stay mute, deaf and blind, right? One sees and has to pretend that hasn’t”* (P4).

Another way of dissociating from the community, at least partially, is using stereotypes to escape crime: *“I use these white headphones a lot, I like them very much. And everyone sees me that way: – oh, hi DJ, hi DJ. So, creating this stereotype, it gets easier to dodge [crime]”* (P7). These stereotypes are related to arts (e.g., music, dance) or religion and in both cases, they serve as a protective shield and also as a divide between us and them groups. Ultimately, participants from the vila reinforce the separation between groups by creating warning crime narratives. These narratives target kids and adolescents aiming at protecting them from violence but above all from getting involved in criminal activities: “*I start to tell [the kids] the story of the people who died [because of violence and drugs], that I have seen, that my wife has seen. The end is only this. This is the end [for criminals]”* (P8).

On the district level, the road between the vila and the neighborhood is a clear representation of the barriers between vila and neighborhood residents. It would have been possible to “cross the road” but neighborhood residents maintain it as a barrier, generation after generation. Residents from the neighborhood have no close interactions with the others from the vila, even though they all had been living there for decades. In this context, neighborhood participants have no interaction with “them”: “*I don’t get mixed, no. Because I think that it is not worth it*” (P11). In contrast to residents from the vila, neighborhood participants are not distancing themselves from the vila residents, they actually just do not interact closely with the others.

In addition, neighborhood participants are othering the ones from the vila, either patronizing “them”*,* belittling "them" in a condescending way or actually even rejecting “them”. For instance, there is rejection associated with the recent urbanization process that changed the entire district landscape but mostly the lives of the ones who lives in the vila: “*The vila got out of them but they haven’t got out … you know. [The urbanization] changed only the appearances, nothing else changed”* (P11). The line between patronizing and rejecting is not always clear and differentiating them function as a way to show different degrees of the othering process.

In summary, the external processes within the vila are more focused on the creation of their own in-group identity in a way that it separates social groups but still focus on coexistence; the processes in the neighborhood on the other hand are more focused on othering. In the first instance, there is a process of breaking connections; in the second, the process is to maintain the distance. The variation in distance between the social groups closely relates to these different processes. Still, both dissociating from the community and maintaining the distance from others lead to the corrosion of social capital.

#### Internal aspects of the construction of social identity

Vila residents are **developing a conflicting self **in a process related to the dissociation from the community. This is because vila residents were up until recently identifying themselves as part of that community. In that sense, vila residents are increasingly disapproving the others’ actions, in relation to the criminal activities and the lack of orderliness and hygiene. However, sharing life stories with “them” make it complex to dissociate from the others and establish their own social identity. For instance, participants have childhood and even family connections with the criminals: “*because the boys [i.e., drug dealers] are my childhood friends”* (P7). They had also shared the favela life, including the perils of the physical and social environments but also the culture, norms and values. Thus, missing the old community life reflects how participants from the vila socially used to identify themselves with the old place: “*I miss the favela a lot, sure. Because it was the place where I was raised in*” (P5).

On the other side of the road, neighborhood residents are currently cultivating a social identity based on the residential area. **Being the neighborhood** is a way to clearly delimitate the us and them in relation to vila and neighborhood*: “From our side here, everything is calm. I don’t see nothing wrong. For me it is all great”* (P3). Participants from the neighborhood perceive differences in the basic infrastructure of the residential area, in the socioeconomic status and culture of the residents and in the different names of the district (referred here as neighborhood A and neighborhood B) in keeping a neighborhood identity: *“What divides neighborhood A is the road. Neighborhood A ends there and from there to up here it is neighborhood B”* (P3). After the urbanization process different names for different parts of the district have been inconsistently used, even by the municipality, also feeding this neighborhood identity discourse.

In the categorization of the vila residents, participants from the neighborhood are stereotyping the vila*,* regarding their choices (or lack of it), values and attitudes: *“It was easier to live in a place where one didn’t pay for water, didn’t pay for electricity … For us from the outside is sad. But for them inside there, it is not. The person relies on the criminals”* (P11). These stereotypes are based on misconceptions but still contributed to the social identification of the ones living on the neighborhood as the others from the vila are portrayed as very different from themselves. Interestingly though, vila and neighborhood residents are not aware that they both identify themselves as humble and worthy*:* “*[Vila residents] are humble people, simple people”* (P5) and “*I don’t have much luxury with things”* (P3) respectively.

Overall with regards to the internal processes, while vila residents are still struggling in developing their self, neighborhood residents are strongly constructing a territorial identification.

### B. "Them" perspectives

In the “them” perspectives groups react and reflect about how they are treated or perceive to be treated by others, irrespectively of their own in-group identification. These perspectives also function as a mirror of the external and internal process of others in higher socioeconomic position while in their “us” perspectives. Still, we should highlight that participants in the “them” perspectives are not passive; they are actually active in their internal and external processes of the construction of social identities that are in this stance marked by protection and preservation.

#### External aspects of the construction of social identity

While vila residents are **safeguarding their own space** within the district, vila and neighborhood residents are** striving to gain influence** in relation to others outside the district. The first is a response to more direct rejection and othering, the latter deals with more complex experiences such as discrimination, segregation and stigmatization. Vila residents as the ones in lower socioeconomic positions face the extra burdens of keeping their space while also striving to gain influence. Neighborhood residents who identify themselves as closer to the others outside the district—which are in higher socioeconomic positions—eventually and notably perceive to be “categorized” by these others as being the same as the ones from the vila.

Vila residents are mainly focused on living their own lives with all its challenges and limitations, trying to thrive and safeguard their space in the district: minding one’s life is enough for them. Still, to deal with the rejection and the stereotypes and in order to protect themselves, participants from the vila needs to downplay the others’ opinions: *“It is horrible. [But] I don’t care. I don’t care anymore, I don’t care” *(P8). Another conceivable way to react in this divided, segregated and above all violent district is to move out if possible: *“lots of people moved out [from the vila], right?”* (P1). Yet, moving out is not always possible or chosen: *“Actually, people don’t move out, because that comfort level one can’t afford in another place. That is why” *(P9).

Different strategies are used by vila and neighborhood residents to strive to gain influence in a context of discrimination, segregation and stigmatization. In pursuing that influence, participants diminish the neighborhood bad reputation, highlighting the positive sides of their places and identities, comparing their district to other districts with similar reputation, even negating reality and normalizing or rationalizing violence: *“Your district is also marked, you can’t say nothing right?”* (P4); and “*It is a district that lots of people discriminate. It is because of this [violence]. But all districts have, nowadays all places have violence*” (P12).

Another very positive alternative to gain influence is to fight for one’s rights*.* Participants are increasingly aware of their rights and managed to gradually get more support from, for instance, the public institutions: “*[my son] is eligible [for special transportation], but before I didn’t know. I was not informed about this. I had the rights and [now] I got it*” (P5). Still the institutional support is not a given and participants describe several occasions in which they had been fighting to access the service and the support they are entitled to: “*I am dealing with the alimony of my kids since 2007 and up until today it ended up in nothing. I have a lawyer but it is not helping*” (P10).

In that search for influence an important aspect is the perceived invisibility of the ones stigmatized. This is especially relevant for residents from the vila in response to the outsiders and also the institutions. They are clearly asking to be seen, exposing the perceptions that people from the outside are too far to actually know who they are and how they live: “*if people entered here once, talked to us and see how it is, the thinking would change completely*” (P2).

Lastly, few participants turn to terrorizing the others in order to gain influence. These experiences although not frequent are clearly an option: “*One sets the terror like this: someone is afraid of me, so I will steal his lunch*” (P1). In that same direction, participants compare the power of crime to having economic power:

“You think no one can touch you, you think that you are the boss, you are the one. It is a good feeling: I can! It is like, I guess it is like a person showing off money. For the criminal it is like that, show off who has more [power], you know” (P8)*.*

### Internal aspects of the construction of social identity

While within the district vila residents **move between shame and pride**, both vila and neighborhood residents **suffer from being unfairly valued**. On the one hand, vila participants feel unwanted in one’s place: *“Even after the construction of the apartment buildings, [residents from the neighborhood] still don’t swallow it, you know. For instance, there was a time that I found out that they wanted to swipe out the [whole] vila”* (P7). At the same time, vila participants are looking alike others while feeling different*:* “*Before it was easy to identify who was from the favela, people had that image of the vila. Nowadays, it is very hard to identify who lives in the vila and who lives in the neighborhood”* (P7); residents from the vila now value how they feel very different from the others from the neighborhood, based on their history and their recent socioeconomic achievements: *“I have a good backpack of experiences because of it, because I have lived here”* (P2). Ultimately, as the overall life of residents from the vila are improving along with progress in economic, infrastructure, housing and access to services, they are increasingly honoring their own achievements*:* “*You become proud. You don’t even want to know anymore, nothing. You are [there]*” (P8).

Residents from the district—both from vila and neighborhood—perceive to be unfairly valued by outsiders including institutions, based on socioeconomic status but mostly on the residential area reputation. Both participants from the vila and from the neighborhood are being discriminated because of the place where they lived. For residents from the neighborhood the discrimination is broadly felt but shifted towards the vila:

“If you live in a district that contains a vila or a favela, big or small, the other districts, let’s say, that don’t have that irregular settlement, they see [here] as the worse way possible. There is a taboo that never ends, that people who lives in the periphery, they are criminals, they are dishonest, they are ill informed, they have no education” (P6)*.*

Participants endure this clear territorial discrimination on top of socioeconomic and racial discriminations: “*There is rejection with you, even to get a job. Should I give the job to this one? Ah, but she lives in the favela, this and that*” (P5). Another woman adds: “*Even more [discrimination] because I am black, right?*” (P3). Discrimination is not limited to the interaction between people but also between people and institutions, such as the police: “*Just knowing we live here in the vila, we are a suspect, or we are already [criminals]*” (P7). Ultimately, participants feel degraded, especially in comparison to others better-off*:*

“Rich people eat well, go wherever they want to. Visits different places … There is a public school that is more like a private one. And it seems [the school] finger pick who they want to study there. You can’t find one single person from [the district] there” (P1)*.*

Interestingly, participants compare themselves to distant references of better-off people, such as people on TV: “*Ah, lots of people on TV is wasting money while I am here, with bills to pay”* (P4).

In trying to make sense of the stigmatization, residents blame themselves for the stigma: “*Because [the bad reputation] is something the vila itself created*” (P7). In that same direction, they also mention the role of media and internet in spreading the bad neighborhood reputation: “*I understand that the outsiders are like this, because one watches bad news reports. One follows the news, one searches, and one finds terrible things*” (P2).

### C. Construction of social identity related to health and social determinants of health

The links between health and the construction of social identities through multiple us and them have been elaborated by the authors based on previous findings and theories. Therefore, it is important to highlight that participants themselves haven’t described or acknowledged this relationship in full, up to health in itself. Still, participants recognize physical and social environments—especially issues associated with crime and violence—as important drivers leading people to get stressed: “*People need to have a peaceful mind. And in the vila, on that matter, it ends up worrying me a bit because of violence, you know”* (P7). Stress is also perceived with the processes of construction of social identities described above.

In addition, residents are getting frustrated with the stigmatization: *“When people talk about favela, about vila, it is like this: it is only ‘favelado’, it is only prostitutes, it is only this, it is only that. It is always to the bad side. I felt frustrated”* (P2). The frustration is greater especially considering residents’ strive to gain influence and their honoring for own achievements. Favelado means a person from the favela. It is a loaded term commonly used in everyday language in urban Brazil, that reduces individuals to the “bad place” where they live.

As already described before, shame and pride and other processes related to the erosion of social capital can also be indicators of the links between the described construction of social identities and health.

## Discussion

This GT analysis allowed for a theoretical understanding of social identity processes involved in the association between contextual factors and known determinants of overall health and well-being. The core category, constructing social identity through multiple us and them, was seen as a relevant aspect for the health inequality debate, considering the variation of the proposed process across social and physical spaces with different levels of social and physical distances with expected consequences for individuals and society. Internal and external aspects of the construction of social identities illustrated and characterized the intersection between self and context, which illuminates the imperceptible everyday practices underlying the production and reproduction of social divisions connected to health inequalities. The present qualitative analysis proposes an interplay of different social determinants of health into a pathway of embodiment [58] through the construction of social identities in a highly unequal urban setting in Brazil. Additional contributions of our study are the characterization of both positive and negative responses—in particular pride and shame—which have been understood in the present analysis as a crucial link between the above-mentioned processes and recognized determinants of health and well-being [59].

Regarding the identification of a given position in the social space, we have found that residents from a vulnerable district in Brazil have used moral and cultural frameworks together with territorial identification as markers of their position. Moral frameworks—i.e., personal characteristics such as humbleness and work ethics—and cultural frameworks—i.e., manners and taste—were more relevant than socioeconomic indicators as intergroup identification criteria, similar to other studies [60]. Regarding the distance from one position to another, it was established, kept and cultivated in ours as well as in others’ studies largely through othering processes based on socioeconomic and territorial markers [46, 59–61].

We found in our study some support to the upward social comparison, in which residents from a vulnerable district compared themselves to references in higher socioeconomic positions, ultimately feeling unfairly valued. These processes and feelings have been previously used to describe the status syndrome that were used to explain the social gradient in health [28]. In contrast to the social comparison theory, the references for social comparison in our analysis were far away—e.g., “rich” people and people on TV—pointing to a choice for distant reference groups [22, 35]. The use of references outside the individuals’ residential area might explain why income inequality have not been associated with health within small-areas [16, 22, 62].

In respect to social stratification and its effects on determinants of health and well-being, we have illustrated a connection of downward othering processes, from positions of immediate as well as of distant higher socioeconomic positions in relation to the socially disadvantaged groups. Previously, downward social comparison has been described as a defense mechanism, in which individuals compare themselves to the ones worse-off attempting to feel better [36]. In a vulnerable district in urban Brazil our findings suggest that othering was actually a source of stress, shame and frustration for the ones othering but mainly for the ones being othered. Both upward and downward social comparisons exposed in the analysis were seen as contributors to the relative deprivation theory, although they illustrate different mechanisms from social position to recognized determinants of health.

Our findings characterize the break of community connections and the lack of interaction between social groups in the same district. The erosion of social capital has been previously associated with poor health in Brazil [10, 63, 64]. There was a shift from community connections to family ties and a turn to private relations and spaces, somehow in contrast to previous research that found stronger community cohesion among the poorest areas in Rio de Janeiro, Brazil [65]. In the present analysis, from an individual level perspective, family ties were described as a source of emotional, financial, instrumental support that can affect several determinants of health; from a collective level perspective, social cohesion is lacking in the community. Pereira and Queiros [49] has also indicated privatization of public spaces, the avoidance of common areas and the elaboration of micro differences in a similar settlement, in Porto, Portugal. Thus, we stress that the predominance of family ties (i.e., bonding social capita) has important implications in the future assessment of social capital and in the interpretation of its effects on health in different settings.

We have demonstrated in the present analysis how neighborhoods through their territorial identification, segregation and stigmatization are powerful components in the articulation and maintenance of social identities and social inequalities. In the stigmatization of these areas, problems are often clustered together—e.g., poverty, racial segregation, unemployment, crime—by the general population, the media and also the state. This feeds the construction of us and them as well as it prevents the development of policies and programs to actually target (i) the most relevant issues of a particular area, for instance crime and violence, and (ii) the structural inequality and marginality behind these problems [50]. Furthermore, our findings support most of the strategies used by residents of stigmatized areas to cope with territorial stigmatization, according to Wacquant, for instance: mutual distancing, retreat into the private sphere, exit, defense of the neighborhood among others [51]. Yet, our findings focused on the role of these strategies in the construction of social identity and on the positive and negative consequences of these strategies for the social comparisons, the erosion of social capital and the developments of pride and shame, which in turn influences social stress and generates or reduces health inequalities. We find this cascade of events helpful to further explain the previously demonstrated association between income inequality, neighborhood basic infrastructure and indicators of social economic position—and its interactions—with health in Brazil [3, 18, 21, 23].

Importantly, we indicate that the construction of social identity through multiple us and them can determine both positive and negative responses at individual level, that in turn have been previously shown to affect health and well-being. On the one hand, it generated frustration, stress and shame. On the other hand, it has also motivated residents from a vulnerable district to fight for their social space and inclusion; to feel proud of their achievements and connected to the place where they live (i.e., emplacement). According to Thomas Scheff [66], developing Erving Goffman’s and C.H. Cooley’s concepts, there is a connection between pride and shame and the social context since these emotions are seen as consequences of self and social processes; they are internal results of external sources. It means that individuals construct a positive or negative view of themselves—e.g., pride or shame—based on correct or incorrect assumptions about how one is viewed by the other(s). Shame for instance has been previously associated with subjective social status, self-rated health and overall health inequalities [59, 67–69]. These positive responses were predominantly at the individual level, lacking direct signs of collaboration or collective action. Nonetheless, as individuals were getting empowered and knowledgeable of their rights, the use and support from institutional services increased, which could be interpreted as linking social capital [70]. The connection between empowerment of individuals with the availability of public services and institutions—e.g., universal health care—supports the positive effects of healthcare and overall social inclusion on the reduction of health inequalities in Brazil [71, 72].

Possible explanations for the perceived lack of connection between contextual factors and health might be related to the participants’ biomedical and individually determined view of health or their notion that health is not in itself a consequence of context but actually a pre-requisite for individuals to do other activities, for instance for working and studying. These health views have been previously described in other settings as well, even though there is important variation regarding countries, socioeconomic conditions, among other factors [73, 74]. Thus, further analysis about these participants’ views on health are considered relevant for health research and will be further elaborated and reported elsewhere.

At last, our findings highlight the relevance of small-area effects above common markers of socioeconomic position—e.g., income, education—in understanding the manifestation of social inequalities in people’s lives. In addition, our findings support the use of multilevel design—including small area levels—in studies about the association between contextual factors and health inequality. Furthermore, in the characterization of these small areas the heterogeneity of the settlement—for instance the presence of vilas and favelas in a particular district—should be used considering that living in a divided district have been shown relevant for health.

### Methodological considerations

The inclusion of participants from both vila and neighborhood side is seen as strength as it allowed us to capture a more comprehensive analysis of the phenomena. We have refrained to make direct comparisons between the groups. Instead we believe they constituted complementary perspectives of the same process. Still, although the number of participants might be considered small, we believe we have enough range, depth and nuances in the data to support our claims, reaching theoretical sufficiency. Further applying Charmaz’ trustworthiness criteria [53], to foster credibility, data collection and data analysis were continuously discussed and reflected upon in the research group. Quotes were used to show how the results are grounded in the data. Our study extends and refines current ideas regarding the effects of context on health, characterizing and connecting the processes and consequences of social identification and othering in highly unequal urban settings, supporting its originality. The study has an intrinsic focus on the links between collectivities and individual lives, which were further articulated and elaborated in the discussion. A limitation regarding resonance is that we haven’t reached the extremes—criminals and extreme poor, neither outsiders and institutions—that could have contributed to portray an even more complete picture of the phenomena. Finally, our findings are deemed useful as it suggests a generic process related to the construction of social identities and its relationships with recognized determinants of health, which develops the health inequality discussion, potentially contributing to future research and policy-making.

## Conclusion

This GT analysis allowed for the characterization of internal and external processes involved in the construction of social identity through multiple us and them in a vulnerable setting in urban Brazil. Our model adds to existing social theories and can be used to increase the understanding of the connections between contextual factors and determinants of health inequalities. Our study highlights the relevance of downward social comparison, territorial segregation and stigmatization and erosion of social capital in the construction of social identities and the manifestation of social hierarchies and neighborhood structures in people’s lives. Ultimately, these create shame and stress but also pride and empowerment, which in turn can affect health and health distribution. The findings present critical implications regarding the design of future research—e.g., socioeconomic position indicators, reference groups for social comparisons, multilevel design and choice of aggregation level—but also the development and implementation of public health/urban programs and policies.

## Data Availability

The datasets generated and/or analysed during the current study are not publicly available due to ethical reasons but are available from the corresponding author on reasonable request.
